# Digestibility of wheat alpha-amylase/trypsin inhibitors using a caricain digestive supplement

**DOI:** 10.3389/fnut.2022.977206

**Published:** 2022-08-10

**Authors:** Angéla Juhász, Mitchell G. Nye-Wood, Gregory J. Tanner, Michelle L. Colgrave

**Affiliations:** ^1^School of Science, Edith Cowan University, Joondalup, WA, Australia; ^2^School of Biosciences, University of Melbourne, Melbourne, VIC, Australia

**Keywords:** alpha-amylase/trypsin inhibitor, proteomics, digestion, prolyl endopeptidase, caricain

## Abstract

Wheat is a major source of nutrition, though in susceptible people it can elicit inappropriate immune responses. Wheat allergy and non-celiac wheat sensitivity are caused by various wheat proteins, including alpha-amylase trypsin inhibitors (ATIs). These proteins, like the gluten proteins which can cause celiac disease, are incompletely digested in the stomach such that immunogenic epitopes reach the lower digestive system where they elicit the undesirable immune response. The only completely effective treatment for these immune reactions is to eliminate the food trigger from the diet, though inadvertent or accidental consumption can still cause debilitating symptoms in susceptible people. One approach used is to prevent the causal proteins from provoking an immune reaction by enhancing their digestion using digestive protease supplements that act in the stomach or intestine, cleaving them to prevent or quench the harmful immune response. In this study, a digestive supplement enriched in caricain, an enzyme naturally present in papaya latex originally designed to act against gluten proteins was assessed for its ability to digest wheat ATIs. The digestion efficiency was quantitatively measured using liquid chromatography-mass spectrometry, including examination of the cleavage sites and the peptide products. The peptide products were measured across a digestion time course under conditions that mimic gastric digestion ***in vivo***, involving the use of pepsin uniquely or in combination with the supplement to test for additive effects. The detection of diverse cleavage sites in the caricain supplement-treated samples suggests the presence of several proteolytic enzymes that act synergistically. Caricain showed rapid action ***in vitro*** against known immunogenic ATIs, indicating its utility for digestion of wheat ATIs in the upper digestive tract.

## Introduction

Wheat is a high-quality source of vitamins, minerals, and protein, though for a subset of the population wheat proteins can elicit one of several enteric immune responses that reduce and threaten health quality. Coeliac disease (CD) affects approximately 1.4% of the population (1), though half of the cases may be undiagnosed (2). In CD, incompletely digested peptides derived from gluten proteins contain specific epitopes (3–5) that initiate an immune response, and a cascade of symptoms leads to inflammation and enteropathy (6). The epitopes that initiate CD are well documented (7, 4, 8) and are typically found in gluten proteins namely gliadins and glutenins. While gluten proteins are established antigens to those with CD and contribute to wheat allergies, non-gluten wheat proteins are potential allergens and antigens capable of causing wheat allergy (WA), baker’s asthma (BA), non-celiac wheat sensitivity is (NCWS), as well as CD (9, 10, 4). Other wheat proteins with a known immune reactive potential include the alpha-amylase/trypsin inhibitor (ATIs) which themselves inhibit proteases and are resistant to digestion. ATIs also contain specific epitopes that can contribute to symptoms in WA, BA (4, 11, 12), and the related disorder wheat-dependent exercise-induced anaphylaxis (13). NCWS occurs when wheat consumption activates the innate immune system and causes enteropathy without serological evidence for both CD and WA (14). Proteins known to be related to NCWS include various subclasses of ATIs (15).

ATIs are members of the prolamin superfamily that share common structural features due to their conserved cysteine skeleton (15, 16) and can be grouped into monomeric, dimeric and chloroform-methanol (CM) soluble proteins (17). ATIs can be detected in most of the cereals, and an immune response against homologous proteins from barley or rye have been confirmed (11, 18). The ATI CM proteins elicit an IgE response in people with BA (19), and typically exhibit inhibitory activity against specific amylases or proteases of plant pests (20, 21). Monomeric and dimeric ATIs are also known to be related to BA while a subclass of ATIs, the dimeric ATI 0.19 proteins, contribute to symptoms associated with CD (22). ATIs may have an inhibitory effect against trypsin and/or amylase from bovines and insects, suggesting a function in plant defense mechanisms. They are digestible by other gastrointestinal proteases including pepsin or chymotrypsin (23), though digestion may release peptides that serve to increase rather than remove the inhibitory activity (24).

There is currently no cure for CD and allergies caused by cereals. The only proven treatment for CD is to avoid the trigger by following a lifelong gluten-free diet. However, given the preponderance of cereals in the modern diet there remains a risk that people inadvertently consume wheat, barley or rye in processed foodstuffs, medications, or in food contaminated during preparation or the supply chain. Various attempts have been made to detoxify gluten and the immune reactive proteins present in cereal products (25), including the use of digestive enzyme supplements that are taken orally and claim to degrade gluten (26). These are an emerging treatment option for inadvertent gluten consumption that directly targets dietary proteins (27). There is an increasing number of commercially available enzyme supplements that typically contain different types of bacterial, fungal or plant proteases. It is important to consider the scientific evidence regarding their efficacy before use.

Papaya latex is a source of enzymes used in the food industry and medicine (28, 29). Crude extract has long been known to digest wheat proteins *ex vivo*, and reduce wheat immunogenicity in susceptible patients (30). It contains the proteases papain, chymopapain, and glutamine cyclotransferase (31, 32), as well as the cysteine protease caricain [EC 3.4.22.30, also known as papaya proteinase omega (33)] which in prior clinical studies has been shown to eliminate the immune reactive proteins leading to a decreased level of immune response (34, 35). It degrades wheat gliadin (36), and recent *in vitro* research has demonstrated that a supplement enriched in caricain was the most effective and rapid enzyme supplement at digesting gliadins (26). This was assessed using enzyme-linked immunosorbent assays that are commercially available and will assess the gluten content but not other immunogenic wheat proteins such as ATIs. While it was primarily designed to detoxify inadvertently consumed dietary gluten, caricain may also be able to reduce the effect of other immune-reactive components in the wheat grain, such as ATIs. To measure the efficacy of the caricain enriched digestive supplement against ATIs, one must either develop and characterize monoclonal antibodies or use an alternative technique such as mass spectrometry (MS), which offers several advantages including higher sensitivity, detecting protein-specific sequence information, and measuring single peptides in a label-free manner. To this end, databases of proteins and epitopes of interest, and sample preparation methods and instrumentation that yield reproducible results exist and enable the comprehensive detection and quantification of immunogenic peptides, before and after *in vitro* digestion.

In this project, we developed proteomics methods to investigate and monitor the digestive effect of a caricain-enriched digestive supplement (GluteGuard) on wheat ATIs to understand its efficacy *in vitro*.

## Materials and methods

### Caricain action on amylase trypsin inhibitors *in vitro*

#### Baseline characterization of amylase trypsin inhibitors in wheat protein extract

To generate protein samples representing the full range of wheat ATIs, samples were generated using an isopropanol-based extraction solvent containing dithiothreitol (DTT) that is known to enrich ATIs (37). Briefly, grain samples from the commercial bread wheat cultivar Baxter were ground into fine powder using a mixer mill (model MM400 Retsch, Germany). Four replicates of 20 mg flour were weighed for each variety, and 10 ml/g of extraction solvent 55% v/v iso-propanol (IPA)/2% w/v DTT (IPA-DTT) was added. Samples were mixed by vortex, incubated for 30 min at 50°C in a thermomixer (Thermo Scientific), and centrifuged for 10 min.

An aliquot (100 μl) of the IPA-DTT extract was applied to a 3 kDa MWCO filter (Merck, Bayswater, Australia), and processed using a filter-aided sample preparation (FASP) method. Briefly, proteins were alkylated using 25 mM iodoacetamide (Sigma, St Louis MO, United States), washed with 25 mM ammonium bicarbonate pH 8.4 (AmBic), before the proteins remaining on the filter were digested using sequencing-grade modified trypsin or chymotrypsin (Promega, Alexandria, Australia) added at a ratio of 50:1 protein-to-enzyme. Digested peptides were collected by centrifugation, washed with AmBic, centrifuged again, and lyophilized in a Speedvac (Thermo Scientific). Dried samples were then reconstituted in 100 μl of 1% formic acid (FA) and subjected to LC-MS/MS.

#### Discovery proteomics

Discovery data was generated using an Eksigent nanoLC415 (Eksigent, Dublin, CA, United States) liquid chromatography system coupled with a TripleTOF 6600 (SCIEX, Redwood City, CA, United States) mass spectrometer operating in data dependent analysis (DDA) mode. In brief, 1 μl of sample was desalted for 5 min on a ChromXP C18 (3 μm, 120Å, 10 × 0.3 mm) trap column at a flow rate of 10 μL/min solvent A and separated on a ChromXP C18 (3 μm, 120Å, 150 mm × 0.3 mm) column at a flow rate of 5 μL/min. The solvents used were (A) 5% DMSO, 0.1% FA, 94.9% water and (B) 5% DMSO, 0.1% FA, 90% acetonitrile, 4.9% water. A linear gradient from 5 to 45% B over 40 min was employed, followed by 45–90% B for 5 min, a 5 min hold at 90% B, return to 5% B over 1 min, and 14 min of re-equilibration. The eluent from the HPLC was directly coupled to the DuoSpray ion source of the TripleTOF 6600 MS. The ion spray voltage was set to 5500 V; the curtain gas was set to 138 kPa (20 psi), and the ion source gas 1 and 2 (GS1 and GS2) were set to 20 and 15 psi, respectively. The heated interface was set to 150°C.

Protein Pilot software v. 5.0.3 with the Paragon algorithm (SCIEX) was used to identify proteins in experimental data. Obtained spectral libraries were searched against a database of Triticeae proteins collected from the Uniprot database (version May 2019) appended with translated gene models obtained from high resolution genome sequencing data of Triticeae species (Ensembl plants), digested *in silico* with trypsin or chymotrypsin as appropriate. ATI proteins were precisely identified in experimental data using conserved protein domain information and manually aligned and analyzed for sub-type annotation using CLC Genomics Workbench v. 21 software platform (Qiagen, Aarhus, Denmark).

#### Measuring rate of enzyme supplement action

Wheat protein extract (15 ml) was generated from Baxter flour using IPA-DTT extraction solution by adding 1.5 mg flour to 50 ml Falcon tubes and adding 15 mL of IPA-DTT solvent. Samples were mixed by vortex, incubated for 60 min on a plate shaker with agitation, and centrifuged for 10 min before the supernatant was stored at -80°C in 7 ml aliquots for the subsequent time-course experiments.

GluteGuard digestive supplements were provided by Glutagen Pty Ltd. Caricain solution was prepared by dissolving one GluteGuard tablet in 9 mL pH 3 acetic acid solution. This was centrifuged and the supernatant diluted 1:9 in 50 mM ammonium bicarbonate pH 7.0 (AmBic) which was diluted a further 10× when added to the reaction vessel in time course experiments. All enzyme solutions were made fresh for each experiment.

Time-course experiments were then performed using caricain after which the wheat protein digests were further cleaved by trypsin, or chymotrypsin (used as experimental tools) to monitor fully tryptic and chymotryptic peptides. Each time course experiment had four replicates containing 1 mL of wheat protein extract and 800 μl of 50 mM AmBic pH 7 as diluent had 200 μl of caricain solution added to it, then 200 μl samples were taken at time points precisely 5, 15, 30, 45, and 60 min after adding caricain. These samples were added to 3 kDa filters containing 200 μl of Stop Solution (pH 3 acetic acid) to shift the pH away from the operational range of caricain (pH 4.5–7.0) and were immediately centrifuged at 20,800 *g* for 15 min. A zero-minute control was made by taking 180 μl wheat protein extract prior to adding caricain and adding to a 3 kDa filter containing 220 μl of Stop Solution. The time-course experiment was performed once for trypsin digestion, and once for chymotrypsin digestion.

The primary filtrate from each time point (Filtrate 1) was collected for digestion specificity analysis. The protein remaining on top of the filter was washed with 100 μl 8 M urea in 0.1M Tris HCl pH 8.4. Reduced cysteine residues were alkylated by adding 100 μl of 25 mM iodoacetamide in 8M urea 0.1 M Tris HCl pH 8.4 and leaving for 20 min in the dark at room temperature. The filter was then centrifuged and washed with AmBic twice to perform buffer replacement. Filters were moved to clean collection tubes, and 200 μl of 20 μg/mL trypsin or alternatively chymotrypsin (Promega, Alexandria, Australia) in AmBic with 1mM CaCl_2_ were added to each filter and the samples incubated overnight at 37°C. Digested samples were centrifuged for 15 minutes at 20,800 g and washed with 200 μl of 50 μl AmBic pH 8.4. The filtrates of digested samples were combined and lyophilized, and peptides reconstituted in 100 μl of 1% FA for later analysis per targeted proteomics as described below.

#### Digestion specificity

Caricain digestion specificity was determined by a detailed sequence analysis using discovery proteomics data taken on the primary filtrate digests. To get this, primary filtrate samples were lyophilized, and reconstituted in 100 μl of 1% FA, then subjected to LC-MS/MS analysis using the same discovery proteomics methods as described above. First, the 60 min time point results from trypsin and chymotrypsin digested extracts were searched using ProteinPilot v. 5.0.3 using the same database as described above with the enzyme setting set to ‘no enzyme’. Peptides identified with > 95% confidence in Protein Pilot were used. The first three (P’1, P’2 and P’3) and last three (P3, P2, P1) amino acid residues were determined for each peptide and the frequency of P’1 – P1 and P’2P’1 – P1P2 pairs calculated.

#### Targeted proteomics

ATI proteins were identified from discovery data using conserved protein domain information obtained from Pfam and InterPro databases and manually aligned for sub-type annotation. Fully tryptic and chymotryptic intense peptides were used to develop multiple reaction monitoring (MRM) methods. Peptides were mapped to the identified sequences using CLC Genomics Workbench v21 and grouped to define ATI sub-type specific peptide sets. MRM transitions were determined for each peptide using the precursor ion and fragment ion m/z values obtained from the discovery proteomics analysis. Pooled samples of the trypsin or chymotrypsin digests were separated on a Exion LC system (SCIEX) and analyzed on a 6500 + QTRAP MS instrument (SCIEX). At least three peptides and four transitions were considered for each protein. From these, three intense transitions with matching peak shape and retention time values were selected for scheduled MRM analysis. Peaks were integrated using Skyline software package (38). The peak areas of the transitions obtained from the same peptide were summed and replicate values were subjected to statistical analysis using GraphPad Prism 8. The resulting peak area measurements were normalized to the 0 min time points, annotated with ATI subtype-specific information, and visualized in the Morpheus R package. Known linear epitopes associated with BA (19) and CD (39) were used for epitope mapping analysis using the Motif search algorithm of CLC Genomics Workbench v. 21 (Qiagen, Aarhus, Denmark).

### Testing the effect of gastric pepsin on the digestive supplement efficiency in non-reducing conditions

Notably, using DTT and alkylation to reduce and stabilize wheat protein cysteine residues will denature wheat proteins and improve protein solubilization during sample preparation, and thereby boost proteome coverage during discovery MS, but it will render proteins more accessible for digestion by a digestive enzyme supplement. To better assess the digestive supplement action under the non-reducing conditions, present in the human digestive system, we adapted the extraction solvent to use 70% ethanol instead of IPA, excluded DTT, and added steps to test the effect of the gastric enzyme pepsin. In this way, proteins were not denatured by reducing conditions and may be more resistant to digestion than in the IPA-DTT extraction. Digestion efficiency of the Gluteguard supplement was compared to a commercially available *Aspergillus niger*-derived prolyl endopeptidase (An-Pep) supplement, containing 360 mg of active ingredient (DSM Nutritional Products SA, Kaiseraugst, Switzerland) per capsule. Experiments using An-Pep were prepared similar to caricain: one capsule was initially dissolved in 9 mL pH 3 acetic acid, before diluting 1:9 in AmBic buffered to pH 3 (the optimum pH of An-Pep) with acetic acid and was further diluted 10x when added to the reaction vessel in time course experiments.

#### Extraction under non-reducing conditions

The commercial bread wheat cultivar Baxter was used in these analyzes. Approximately 3 g flour was weighed into eight 50 ml Falcon tubes and proteins extracted by adding 10 ml/g 70% ethanol in non-reducing conditions. Flour samples were vortexed thoroughly, before being sonicated for 15 min and vortexed again. They were then mixed for 30 min on a plate shaker operating at 600 rpm and centrifuged at 4,500 rpm for 10 min to form a pellet. The tubes were then decanted into a single flask, and aliquoted and stored at -80°C until needed. The protein concentration in this extract was measured at 2.07 mg/ml using a Bradford assay (Sigma) prior to freezing. Working caricain and An-Pep solutions were made fresh on the day of use as described above.

For caricain experiments, a digestion time course experiment was designed with an initial pepsin step to simulate the gastric phase of digestion. The gastric pre-treatment step consisted of four replicate reaction vessels containing 280 μl of water, 100 μl of 1% HCl, 1 ml of protein extract solution, and either 20 μl of 200 μg/ml pepsin in acetic acid pH 3 or 20 μl of acetic acid pH 3 as a control. These were combined and digested for 60 min at 37°C, after which 140 μl was taken as the 0 min time point sample and added to 3 kDa filters that were pre-loaded with 260 μl AmBic pH 8.4 to quench pepsin activity. To the reaction vessel 600 μl of AmBic pH 8.4 was added to neutralize acid and bring pH to 7, quenching pepsin activity. The reaction vessel was then vortexed, 200 μl of working caricain solution was added, vortexed again, and samples (200 μl) were collected from the reaction vessel at time points precisely 1, 5, 15, 30, 45, and 60 min after adding caricain solution. The 1-minute sampling time point was added to capture rapid digestion suggested by the experiments above. Samples taken at each time point were applied to 3 kDa filters that were pre-loaded with 200 μl pH 3 acetic acid to quench enzyme activity and were centrifuged immediately at 20,800 g for 15 min. Samples were subjected to a FASP in a protocol that excluded reduction and alkylation steps such that the subsequent enzymatic digestion (using trypsin, and in independent experiments chymotrypsin) better reflected digestion in the digestive tract *in vivo*. Control experiments were also included where caricain was excluded and substituted for blank solution (50 mM AmBic pH 7). In this way and *in vitro* modeling of ATI digestion was tested, and experiments with and without pepsin, with and without caricain, and with trypsin or chymotrypsin used in FASP for analytical purposes inform our understanding of caricain action *in vivo*. Combinations of enzymes in these experiments are shown in Supplementary Table 1.

The time course experiment was adapted to be used with An-Pep, which is active in the low pH environment of the stomach. The time course sampling was performed throughout the 60 min gastric digestion step, and there was no intestinal digestion. Four replicate reaction vessels each contained 1 ml protein extract solution, 780 μl of 1% HCl as diluent, and either 20 μl of 200 μg/ml pepsin in 1% HCl or 20 μl of blank 1% HCl. Vessels were vortexed and a 178 μl time point sample was taken immediately and added to 3 kDa filters containing AmBic pH 8.4 to quench enzyme activity, and centrifuged. To the reaction vessel either 200 μl An-Pep or 200 μl acetic acid were added and samples collected after 1, 5, 15, 30, 45, and 60 min, and added to filters containing AmBic pH 8.4, centrifuged, and processed by FASP again excluding reduction and alkylation steps. There were experiments run with and without pepsin, with and without An-Pep, and with trypsin or chymotrypsin used in FASP as summarized in Supplementary Table 1.

## Results

### Characterizing extractable wheat amylase trypsin inhibitors

**FIGURE 1:**
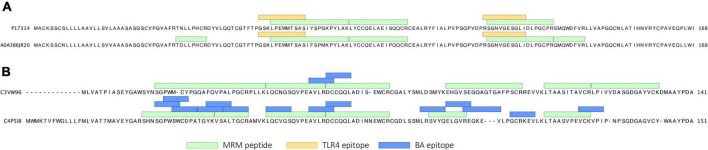
**(A)** Two ATI CM3 protein isoforms (P17314 and A0A3B6JR20) detected in the flour samples with TLR4 epitopes and monitored MRM peptide sequences flagged. **(B)** Polypeptide sequence of representative dimeric (C3VW96) and monomeric (C4P5I8) ATI proteins, with BA epitopes and peptide sequences targeted for quantification.

In the initial discovery proteomics experiment, 265 proteins were identified from IPA/DTT extracts of cultivar Baxter. Of these proteins, 22 ATI isoforms were identified. The precise characterization of the ATI sequences resulted in nine ATI subtypes, including six CM ATIs (CM1, CM2, CM3, CM16, CM17, CM Hageman), monomeric ATIs, and two sub-types of dimeric ATIs (dimeric-0.19 and -0.53 ATIs) (Supplementary Table 2). Epitope mapping analyzes confirmed the presence of the TLR4 epitopes (39) in sequences from the CM3 ATI sub-type and BA-related B-cell epitopes (19, 39) in the dimeric and monomeric ATI sequences (Supplementary Table 2). Using peptide mapping with 100% sequence identity we categorized the quantified peptides into sets that are specific for the following major ATI types: dimeric, dimeric-0.19, monomeric, CM3, CM16-CM17 and CM1 ATIs. Peptides overlapping with published CD (39) or BA associated epitopes (19) were included in the analysis. A total of 69 fully tryptic peptides identified from the ATI proteins were monitored using LC-MRM-MS. Figure 1 shows two identified CM3 sequences with peptides known to be related to the TLR4 response (39). These TLR4 epitopes were partially overlapping with fully tryptic MRM peptides. Epitopes associated with BA were detected in the monomeric and dimeric ATIs.

### Enzymatic digestion time course

**FIGURE 2:**
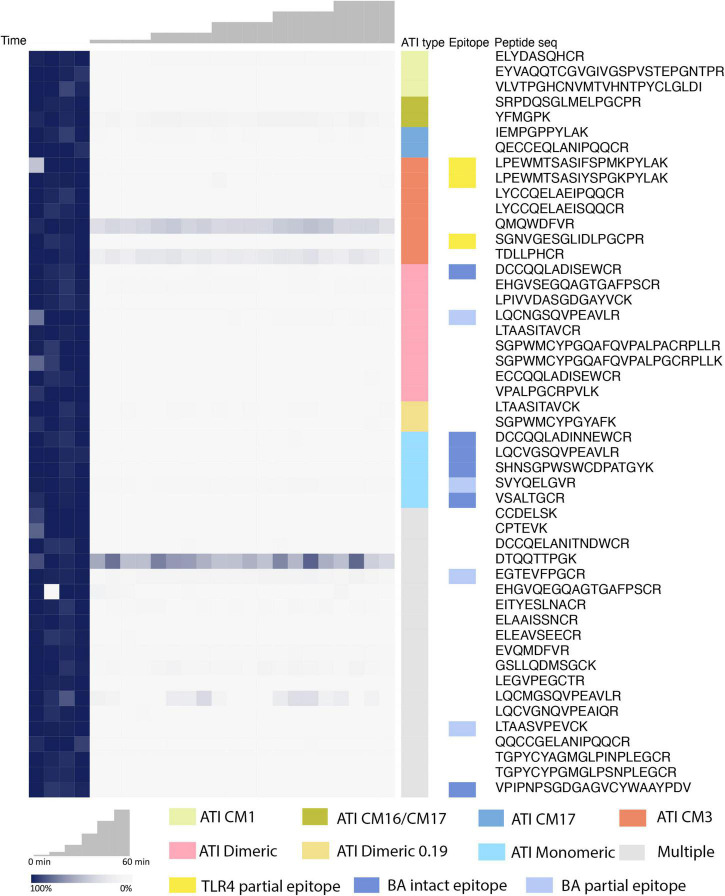
Overall trends of ATI peptide digestibility in Baxter. Peptide peak area values of all peptides included in the analysis are presented in the individual replicates and monitored during the digestion analysis. Majority of the peptides were more than 95% reduced within 5 min of digestion, including those present in one or more subtypes of ATIs that are immune-reactive.

Targeted proteomics was used to quantify fully tryptic and chymotryptic ATI peptides in undigested wheat extracts (0 min time point), and the relative abundance after digestion by caricain for 5, 15, 30, 45, and 60 min was measured. Practically, this was achieved by using trypsin or chymotrypsin as a biochemical tool to liberate detectable peptides during FASP that would be suitable for MS measurement. Peptide peak areas were recorded for each MRM transition in Baxter samples, each peptide was normalized to its’ baseline peak area at 0 min, and abundance values visualized as a heatmap (Figure 2).

Comparison of the ATI subtypes show all subtypes have been successfully digested by caricain within 5 min (Figure 2). This includes multiple ATI subtypes known to exhibit immune reactivity, as presented in turn in the following sections.

### Abundance changes in the amylase trypsin inhibitor subtypes with known immune reactivity

Altogether eight dimeric ATI isoforms were detected, one of which represents the CD-associated dimeric-0.19 ATI subtype. Most of the dimeric ATI isoforms contained two epitopes associated with BA (19), and the monomeric ATI isoforms contained 14-15 BA epitopes (Supplementary Table 2). We were able to detect several caricain-specific cleavage sites both within and around the epitopes (Supplementary Figure 1) and were able to monitor the abundance of peptides that span their sequence by LC-MRM-MS (Figure 1). In all cases, these peptides were present prior to caricain action, but dramatically decreased in abundance within 5 min (Figure 2).

Dimeric-0.19 ATIs were represented by the protein isoforms Q5UHH6 and Q5UHH8. Compared to dimeric ATIs one peptide, ECCQQLADISEWCR, was identified that was specific for this sub-type and two peptides, SGPWMCYPGYAFK and LTAASITAVCK, were shared between dimeric-0.19 and -0.53 ATIs. Interestingly, the ECCQQLADISEWCR peptide could only be measured at 0 min and presumably completely digested in later time points.

**FIGURE 3:**
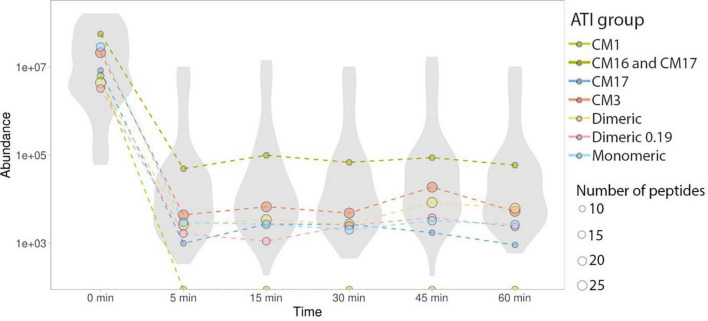
Abundance changes of ATI type-specific peptides. In all detectable ATI subtypes, peptide abundance (reported as MRM peak area, log scale) decreased by orders of magnitude within 5 min and any change in abundance that occurred after this time was minimal. Median values of ATI subtype-specific peptide groups are labeled with colored dots. Dot size represents the number of peptides, sub-type specific data distribution and heterogeneity is highlighted using violin plots.

Monomeric ATIs are enriched in peptides that can trigger immune response in BA. There were four distinct isoforms of monomeric ATIs identified. All monitored peptide sequences showed a significant decrease in peptide abundance in the early stages of protein digestion (Figures 2, 3).

There were two CM3 ATI protein isoforms detected, that differ in their protein sequences (P17314 and A0A3B6JR20). Both proteins include the TLR4 epitopes reported by Cuccioloni et al. (39) and fully tryptic peptides overlapping with these epitopes following caricain digestion have been identified in our analysis (Figure 1 and Supplementary Figure 1). All the CM3 peptides show a significant decrease in their abundance after 5 min of digestion. Monitoring peptides overlapping with the epitopes demonstrate that caricain efficiently cleaves these epitopes within 5 min (Figures 2, 3).

### Digestion specificity

Peptide sequences detected in the F1 filtrates were used to determine the cleavage specificity of enzymes present in the GluteGuard digestive supplement. Combinations of amino acid residues present in P3, P2, P1 and P1’, P2’, P3’ positions of the detected peptides detected from ATI protein sequences were analyzed. Detected primary filtrate peptide sequences were mapped to sequences using 100% sequence identity cut off, and extended peptides, with 3 amino acid flanking regions in both directions were extracted from the complete sequence data, and further analyzed.

**FIGURE 4:**
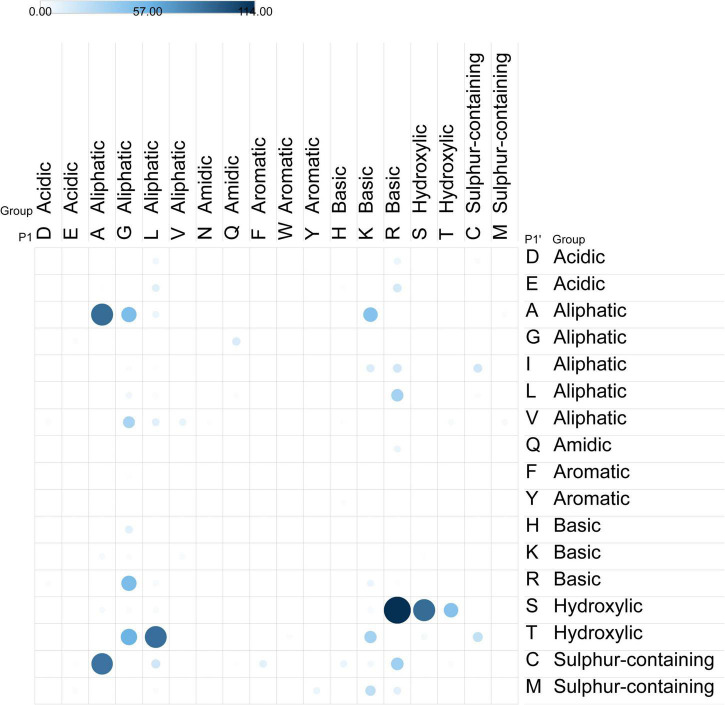
Cleavage sites observed in caricain-digested peptides. Dot color and size indicates frequency of observed P1-P1’ pairs in primary filtrate peptides. The observed caricain cleavage sites demonstrate preferential cleavage between aliphatic residues at R-S, followed by A-A, S-S, L-T, and A-C.

The most frequent combination was when the sequences were cleaved before (N-terminal) an aliphatic amino acid in all P1’, P2’ and P3’ positions combined with cleavage after (C-terminal) an aliphatic in P3, hydroxylic in P2 and sulfur-containing in P1. When only the P1, P2 and P1’, P2’ positions are considered the most frequent combinations mainly contain aliphatic residues in the P1’ and P2’ positions, where P1’ is mostly A or V. This is combined with aliphatic residues present in the P1 position, with glycine being the most frequent residue. The ATI-specific caricain cleavage patterns confirm the importance of aliphatic residues both in P1’ and P2’ positions (Figure 4).

### Impact of gastric pretreatment on digestion efficiency

The 69 fully tryptic and chymotryptic ATI peptides monitored previously were then searched for in samples extracted and digested under non-reducing conditions. The additional effect of the gastric enzyme pepsin on ATI digestion was also quantified, to better reflect the human digestive system.

Of the 69 peptides in the previous MRM transition list, 47 were detected in samples extracted with 70% ethanol as extraction solvent at sufficient levels for quantitation. These peptides primarily represent the CM sub-class of ATIs (CM1, CM3, CM16, and CM17), while monomeric and dimeric ATI-specific peptides were under-represented. Most of the peptides showing good intensities were those lacking cysteine residues in their sequence, and those with cysteine were generally below the threshold intensity. As the protein digestion workflow did not incorporate reduction or alkylation, Cys-containing peptides may contain disulfide linkages which would preclude their detection or identification.

**FIGURE 5:**
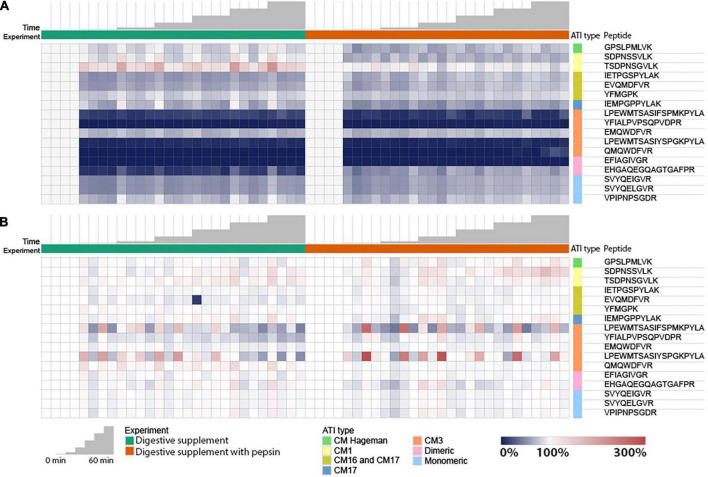
Relative abundance changes of the tryptic ATI peptides digested by: caricain **(A)**; and An-Pep **(B)**; in non-reducing conditions in absence (left) and presence (right) of pepsin. Each value (peptide peak area) was normalized to its own 0 time point value.

To account for differences in initial abundance, experiments with caricain alone (Ccn) and caricain pre-treated with pepsin (Ccn + Pep) had peptide abundance values normalized to their 0 min time point value. The heatmaps below show these 0-normalized abundance results, where blue shades indicate a decrease, red an increase, and white a constant value (Figure 5A). Similar assessments were made of An-Pep alone (An-Pep) and with pepsin pre-treatment (An-Pep + Pep) (Figure 5B).

The majority of monitored peptides were digested by caricain under non-reducing conditions (Figure 5A). Despite collecting samples at a time point 1 min after adding caricain, the change between 0 and 1 min was generally greater than the change between 1 and 60 min. Peptides from CM3, CM16/17, dimeric ATIs, and to lesser of an extent monomeric ATIs and CM Hageman ATIs decreased in abundance within 1 min of digestion with caricain. Of note, peptide TSDPNSGVLK specific to the ATI CM1 type showed an increase in abundance in the caricain time course experiment, though this was not seen when pepsin was used to simulate gastric digestion. This shows that caricain alone leads to incomplete digestion of CM1 proteins, though when acting in conjunction with pepsin digestion is complete.

**FIGURE 6:**
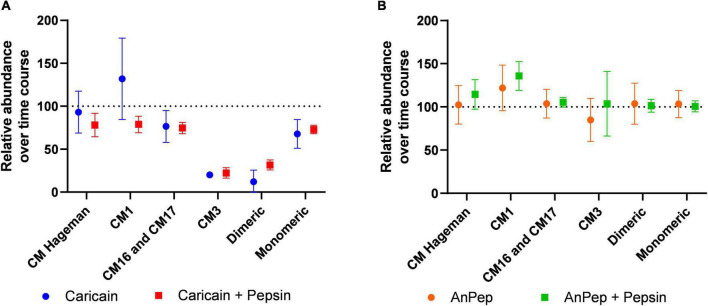
Peptide abundance after 60 min digestion expressed as a percentage of abundance in the 0 min time point. ATI CM16 and CM17 peptides showed similar results and are grouped together **(A)** caricain significantly digests all ATI types except for CM Hageman and CM1, though in conjunction with pepsin, caricain digests these too. **(B)** An-Pep does not digest ATI peptides within 60 min even in conjunction with pepsin. Error bars indicate 95% confidence intervals.

The mean relative change in peptide abundance over 60 min was calculated for each ATI-specific peptide group for caricain, and caricain pre-treated with pepsin (Figure 6A). The highest decrease in abundance (> 80%) was measured in the dimeric ATI and ATI CM3 peptides, which indicates that the main immunogenic ATIs can be degraded by this digestive supplement. All ATI types are digested by caricain to different degrees. The CM16, CM17 and monomeric group specific peptides were only reduced by 20–30%, and the overall average abundance of ATI peptides after 60 min of digestion was 59.7% that of their abundance in the 0 min control. The presence of pepsin did not significantly affect peptide digestibility except in the case of CM1 peptides, where it significantly reduced abundance. Digestion by An-Pep in the presence and absence of pepsin confirmed that An-Pep is not able to digest ATIs effectively (Figure 6B).

## Discussion

In this study, we investigated the efficiency of a digestive supplement enriched in caricain at digesting ATIs, a protein group with known immune reactive potential in CD, food allergy and NCWS. Under both reducing and non-reducing conditions, caricain rapidly digested ATIs including the most immunologically relevant ATI subtypes. With one exception, multiple ATI proteins belonging to the dimeric, monomeric, CM1, and CM3 subfamilies significantly decreased within 1 min, leaving little signal at the 5 min and later time points. This rapid action is similar to recent observations of caricain action on wheat gliadin proteins (26). While it is possible that caricain is not completely inactivated by the addition of pH 3 stop solution, the large relative change between 0–5 min and the lack of change between 5–60 min would reinforce the conclusion that the enzyme acts rapidly *in vitro*, in conditions that resemble human digestion. The sole exception was the CM1 peptide TSDPNSGVLK in non-reducing conditions, which uniquely increased in abundance with caricain. This is suggestive of structural changes in the parent protein(s) instigated by caricain, which liberates previously hidden protein regions such that they are amenable to digestion by other digestive enzymes. Importantly, when pepsin was used to simulate gastric digestion the increase in TSDPNSGVLK abundance was not noted, suggesting that the combination of pepsin and caricain improved the digestibility of this protein in our *in vitro* model.

The ATI identification rate is improved when proteins are reduced during extraction. This is not surprising given that ATIs exhibit trypsin inhibition, and their secondary structure is stabilized by disulfide bridges, and it is consistent that reduction and alkylation will both aid their extraction and cause denaturation, removing their trypsin inhibitory activity (16) and improving digestion by trypsin and chymotrypsin. In non-reducing conditions, however, the stable conserved structure of ATIs contributes to their poor digestibility and means they can reach the intestine intact where they are able to bind to the TLR4 receptor and instigate an immune response. Our results suggest that caricain effectively cleaves most ATI peptides including immune reactive regions related to the TLR4 response or BA and facilitates further digestion by intestinal proteases including trypsin and chymotrypsin.

Comparing the protein extracts derived under experimental (IPA/DTT) and simulated gastric (i.e., extracted in 70% ethanol under non-reducing conditions) revealed that some proteins were solubilized/extracted in IPA/DTT but not in the ethanol solutions used. Those that were extracted and hence detectable showed different responses to caricain according to the ATI subtype they belong to, which in turn suggests that some ATI types exhibit a resistance to digestion, perhaps due to hinderance in structures stabilized by disulfide bridges. It is likely that protein regions buried due to the compact disulfide bridge stabilized structure of ATIs makes them less prone to digestion, and contributes to their ability to allergic reactions (39). Notably, the inclusion of a gastric phase, practically implemented through use of pepsin, did not enhance digestion.

An-Pep is another commercially available digestive supplement for gluten-sensitive consumers, designed to act upon gliadins. This test of its action against wheat ATIs revealed limited An-Pep efficacy against some but not all ATI proteins under non-reducing conditions, showing an overall lower efficacy when compared to caricain. While ATI CM17 proteins decreased in abundance after An-Pep digestion, CM1 peptides were more abundant when pepsin is also present (Figure 6). This digestion ability of An-Pep was not improved by the presence of pepsin.

The varied digestion specificity analysis of caricain-enriched digestive supplement (Figure 4) indicates that there is more than one enzyme contained in the papaya latex extract. While gluten protein digestion by this supplement has been attributed to the enzyme caricain (26), this is the first assessment of ATI digestion by this supplement. Most cleavages observed in primary filtrate peptides occurred where aliphatic residues are in both the P1’ and P1 positions, though this is more variability than would be expected from a single enzyme. The most common cleavage sites were R-S, followed by A-A, S-S, L-T, and A-C, though more were also observed (Figure 4). The number of glycine residues may account for some of the G-A and G-V cleavages. The papaya protease papain (EC 3.4.22.2) cleaves after R or K residues and before hydrophobic residues, and would explain the R-S, as well as K-A and R-L activity observed. The enzyme caricain (EC 3.4.22.30) is reported to have broad specificity for peptide bonds like papain (EC 3.4.22.2) and chymopapain (EC 3.4.22.6). Future directions in this line of research might focus on identifying the enzymes that cleave specifically at A-C: while a tablet with multiple protease cleavage sites is evidently effective at denaturing and digesting most ATIs, a more concentrated protease may prove more potent against those that resist digestion.

## Data availability statement

The datasets presented in this study can be found in online repositories. The names of the repository/repositories and accession number(s) can be found below: http://www.proteomexchange.org/, PXD034720.

## Author contributions

AJ: project design, experimental design, sample preparation, data analysis, and manuscript preparation. MN-W: experimental design, sample preparation, LC-MS data collection, data analysis, and manuscript preparation. GT: project design and provision of materials. MC: project design, experimental design, data interpretation, and manuscript preparation. All authors contributed to the article and approved the submitted version.
