# Supplementary material for: Digestibility of wheat alpha-amylase/trypsin inhibitors using a caricain digestive supplement
Source: Front Nutr. 2022 Aug 10;9:977206. doi: 10.3389/fnut.2022.977206 (PMC9399795; doi:10.3389/fnut.2022.977206)
Supplement: Supplementary file 2 [file Table_2.DOCX]

**Supplementary Table 1:** Breakdown of enzyme combinations used in time course experiments (E#), using the extracts without DTT. In caricain experiments, time course samples are collected after caricain addition in the intestinal digestion steps. In AnPep experiments, time course samples are collected after AnPep addition in the gastric digestion steps. The presence or absence of the enzyme is indicated by Y (yes) or N (no).

| E# | Gastric digestion | | Intestinal digestion | Analytical digestion |
| --- | --- | --- | --- | --- |
|  | Pepsin | AnPep | Caricain | Trypsin (T) or Chymotrypsin (C) |
| 1 | N | N | Y | T |
| 2 | Y | N | Y | T |
| 3 | N | N | Y | C |
| 4 | Y | N | Y | C |
| 5 | N | Y | N | T |
| 6 | Y | Y | N | T |
| 7 | N | Y | N | C |
| 8 | Y | Y | N | C |
