# Supplementary figures and images for: Digestibility of wheat alpha-amylase/trypsin inhibitors using a caricain digestive supplement
Source: Front Nutr. 2022 Aug 10;9:977206. doi: 10.3389/fnut.2022.977206 (PMC9399795; doi:10.3389/fnut.2022.977206)

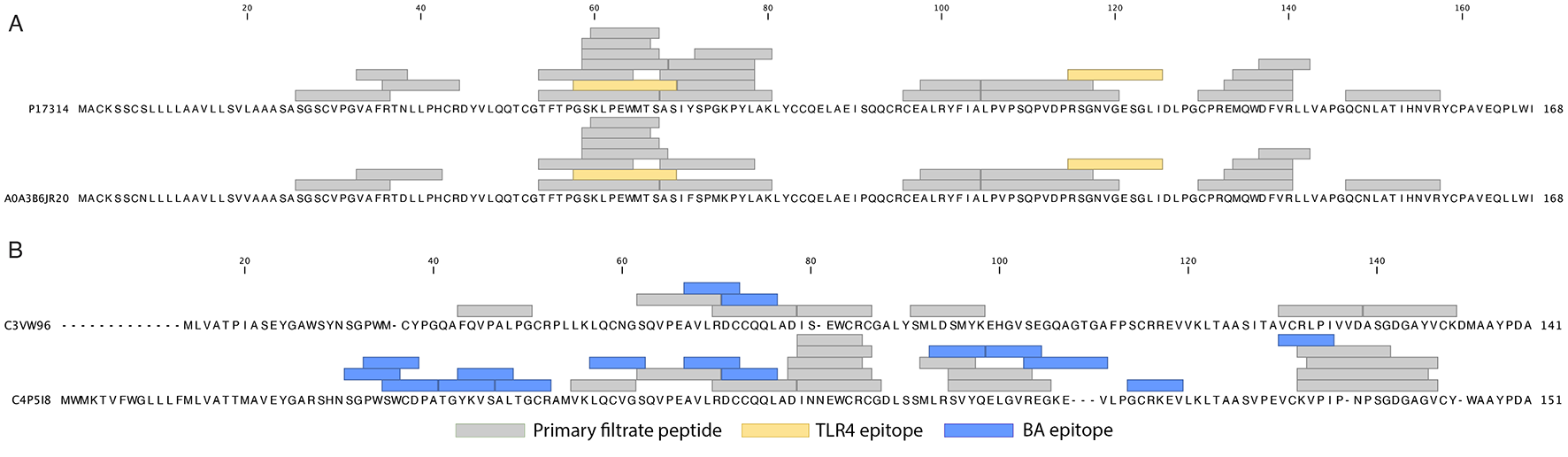

Supplement: Supplementary file 3 [file Image_1.TIF]
